# The Operations of Surgery

**Published:** 1889-03

**Authors:** 


*The Operations of Surgery*. By W. H. A. JACOBSON, F.R.C.S. Pp. 1136. London: J. & A. CHURCHILL. 1889.

To gather within the limits of one volume, however ponderous, an adequate account of modern operative surgery seems an almost impossible effort. Indeed, according to the ordinary limits of descriptive language, detailed and exhaustive accounts of all surgical operations could not be given in anything smaller than a cyclopædia. And such a detailed and exhaustive work could not be written by one man, for here experience would be essential, and few surgeons, from experience, are competent to speak authoritatively on more than two or three operations.

The task which Mr. Jacobson has set himself to perform is, while not of this exhaustive and encyclopædic nature, more ambitious than most men, single-handed, have dared to undertake. He traverses the whole range of operative surgery. For each operation he provides a description which, to the ordinary handicraftsman educated in anatomy and the principles of surgery, is sufficiently complete for work-a-day purposes. In language, method, and illustration the work is in every respect admirable. We consider, therefore, that Mr. Jacobson has succeeded in the task he has set himself, and that he has produced a work on operative surgery which, in width of scope and fulness of detail, has scarcely a rival in the English language. With judicious trimming, careful husbanding, and constant embellishment as the art of surgery advances, this work should hold a commanding position for years to come.

In respects not specially professional, the author has shown himself expert in scientific book-making. There is an excellent index; the arrangement and balance of material is simple, judicious, and harmonious; the language is clear and elegant; and the grammar and rhetoric are (rare observation in these days!) above reproach.

